# Space radiation quality factor for Galactic Cosmic Rays and typical space mission scenarios using a microdosimetric approach

**DOI:** 10.1007/s00411-023-01023-6

**Published:** 2023-04-16

**Authors:** Alexis Papadopoulos, Ioanna Kyriakou, Sébastien Incerti, Giovanni Santin, Petteri Nieminen, Ioannis A. Daglis, Weibo Li, Dimitris Emfietzoglou

**Affiliations:** 1 Medical Physics Laboratory, Department of Medicine, University of Ioannina, 45110 Ioannina, Greece; 2 University of Bordeaux, CNRS, LP2I, UMR 5797, F-33170 Gradignan, France; 3 ESA/ESTEC Space Environments and Effects Section, ESTEC, Keplerlaan 1, 2200 AG Noordwijk, ZH The Netherlands; 4 Department of Physics, National and Kapodistrian University of Athens, 15784 Athens, Greece; 5 Hellenic Space Center, 15231 Athens, Greece; 6 Helmholtz Zentrum München, German Research Center for Environmental Health (GmbH), 85764 Neuherberg, Germany; 7 Present Address: Federal Office for Radiation Protection (BfS), Ingolstädter Landstraße 1, 85764 Oberschleißheim, Germany

**Keywords:** Space radiation, Quality factor, Galactic Cosmic Rays, Manned space missions, Carcinogenic risk, Microdosimetry

## Abstract

Space radiation exposure from omnipresent Galactic Cosmic Rays (GCRs) in interplanetary space poses a serious carcinogenic risk to astronauts due to the—limited or absent—protective effect of the Earth’s magnetosphere and, in particular, the terrestrial atmosphere. The radiation risk is directly influenced by the quality of the radiation, i.e., its pattern of energy deposition at the micron/DNA scale. For stochastic biological effects, radiation quality is described by the quality factor, $Q$, which can be defined as a function of Linear Energy Transfer (LET) or the microdosimetric lineal energy ($y$). In the present work, the average $Q$ of GCR for different mission scenarios was calculated using a modified version of the microdosimetric Theory of Dual Radiation Action (TDRA). NASA’s OLTARIS platform was utilized to generate the radiation environment behind different aluminum shielding (0–30 g/cm^2^) for a typical mission scenario in low-earth orbit (LEO) and in deep space. The microdosimetric lineal energy spectra of ions ($Z\ge 1$) in 1 μm liquid water spheres were calculated by a generalized analytical model which considers energy-loss fluctuations and δ-ray transport inside the irradiated medium. The present TDRA-based $Q$-values for the LEO and deep space missions were found to differ by up to 10% and 14% from the corresponding ICRP-based $Q$-values and up to 3% and 6% from NASA’s $Q$-model. In addition, they were found to be in good agreement with the $Q$-values measured in the International Space Station (ISS) and by the Mars Science Laboratory (MSL) Radiation Assessment Detector (RAD) which represent, respectively, a LEO and deep space orbit.

## Introduction

Depending on radiation dose, space radiation exposure may pose a health risk to astronauts that intend to travel in interplanetary space, to Mars or the Moon. Among other space hazards that astronauts will face in long-duration space mission, radiation is considered of paramount importance (National Research Council 1997; National Council on Radiation Protection and Measurements 2000, 2006; Cucinotta et al. 2002, 2013; Board and National Research Council 2006; Durante and Cucinotta 2008, 2011; Durante 2014). The space radiation environment is quite different from that encountered on the surface of Earth, which are mostly *X* and *γ* rays and a small component of alpha particles from (mainly) radon (National Council on Radiation Protection and Measurements 2001, 2009; International Commission on Radiological Protection 2007; Restier-Verlet et al. 2021). The space radiation field is composed of highly energetic ions of a wide range of atomic numbers. It includes the constant and isotropic Galactic Cosmic Rays (GCRs), the sporadic Solar Particle Events (SPE), and the Van Allen belts (VA) in the Earth’s magnetosphere (Reitz 2008; Chancellor et al. 2021).

GCR consists of a baryon (98%) and an electron component (2%). Baryons include protons (~ 85%), alpha particles (~ 14%) and high-atomic number, and high-energy Ions (HZE) up to uranium (~ 1%), with energies peaking around GeV/amu, while reaching up to ~ TeV/amu and beyond. The most probable source of these particles are high-energy phenomena from supernova blast waves (< ${10}^{15}\mathrm{eV}$) or even neutron stars (Reitz 2008; LAT Collaboration et al. 2013; Blasi 2013; Moraal 2014). The solar cycle can affect space mission planning, by decreasing (at solar maximum) or increasing (at solar minimum) the absorbed doses from GCR that astronauts receive (O’Neill 2006; Reitz 2008; LAT Collaboration et al. 2013). The GCR effective dose rates in deep space, although ~ 1000 times greater than on Earth, are considered relatively low and do not cause acute health effects. As a result, the biological concerns from GCRs are mostly carcinogenesis and the degenerative late effects of specific tissues, such as cardiovascular disease (CVD), damage to the Central Nervous System (CNS), and the induction of cataracts (National Council on Radiation Protection and Measurements 2006; Durante and Cucinotta 2008; Maalouf et al. 2011; Dietz et al. 2013; Semkova et al. 2014; Kennedy 2014; Freese et al. 2016; Elgart et al. 2018; Mitchell et al. 2019; Meerman et al. 2021; Tinganelli et al. 2021; Reynolds et al. 2021; Cortés-Sánchez et al. 2021). However, the magnitude and the different types of biological effects caused by HZE particles from GCR are not fully known (National Research Council 1997; National Council on Radiation Protection and Measurements 2000; Durante et al. 2001; Durante 2004; Cucinotta and Durante 2006; Durante and Cucinotta 2008, 2011; International Commission on Radiological Protection 2013; McKenna-Lawlor et al. 2014; Cucinotta et al. 2020b; Cucinotta 2022). In LEO orbit (such as on the International Space Station, ISS), the effective dose rates from GCR are lower than in deep space by a factor of about 2, due to the additional shielding from the Earth’s magnetic field, although this is dependent on the inclination and the altitude of the mission (Benton and Benton 2001; Reitz 2008; Semkova et al. 2014). For more detailed information about the radiation environment and radiation dosimetry in LEO, the readers are referred to the literature (International Commission on Radiological Protection 2013; Badhwar et al. 1992b, a, 1994, 1995, 1996, 1992b, 1998, 2002; Golightly et al. 1994; Badhwar 1997, 1999, 2000, 2002; Badhwar and Cucinotta 1998; Doke et al. 2001b, a; Benton et al. 2002; Akopova et al. 2005; Bartlett et al. 2006; Zhou et al. 2007; Cucinotta et al. 2008; Berger 2008; Reitz et al. 2009; Straube et al. 2010; Narici et al. 2015; Dachev et al. 2017).

Radiation quality depends upon the type and energy of the particle and has been linked to the induced biological effects caused by the distinct energy-deposition pattern. Radiation quality is commonly described by the relative biological effectiveness (RBE), which for stochastic effects (mainly carcinogenesis) at low doses is termed quality factor, $$Q$$. Quality factor ($$Q)$$ can be calculated as a function of the non-stochastic unrestricted Linear Energy Transfer (LET), i.e., the mean electronic energy loss of a charged particle per unit path length (International Commission on Radiological Protection 1991; International Commission on Radiation Units and Measurements 1986), which is currently adopted by the European Space Agency (ESA) and the Canadian Space Agency (CSA) for the estimation of the carcinogenic risk of astronauts in space missions. This approach, however, has some noteworthy drawbacks for space operations (National Council on Radiation Protection and Measurements 2001; Goodhead 2018). On the contrary, microdosimetric approaches describe the radiation quality through the stochastic analog of LET, lineal energy ($$y$$) (International Commission on Radiation Units and Measurements 1986). The microdosimetric quality factor ($$Q$$) can be calculated via $$y$$ using the ICRU Report 40 (Joint Task Group on Radiation Protection Quantities et al. 1986) methodology or the Theory of Dual Radiation Action (TDRA) (Kellerer and Rossi 1978; Kellerer 1985; Rossi and Zaider 2012). Theoretical calculations of $$y$$-spectra for ions relevant to GCR, require specialized Monte-Carlo codes (e.g., GEANT-4 DNA, PHITS, PARTRAC, KURBUC) (Incerti et al. 2010; Alloni et al. 2012; Liamsuwan et al. 2014; Nikjoo et al. 2016; Matsuya et al. 2021, 2022) or analytical microdosimetric models. Such a model has been developed by Xapsos (Xapsos et al. 1994, 1996; Badavi et al. 2009; Papadopoulos et al. 2022), in order to calculate the ion’s $$y$$-spectrum in nano- to micro-meter targets based on LET, considering energy-loss straggling parameters and additional corrections of the finite range of δ-rays.

NASA has developed its own approach for determining $Q$, which differs from both ICRP (Publication 60) and ICRU (Report 40) recommendations. NASA’s quality factor is a function not only of LET, but also of the atomic number ($Z$) and the velocity ($\beta$) of the particle via the track-structure parameter ${Z}^{2}/{\beta }^{2}$ (Cucinotta et al. 2011, 2013; Council 2012; Goodhead 2018).

The scope of the present work is to: (i) Calculate the GCR spectrum (1 MeV/amu—1 GeV/amu) behind aluminum shielding (0–30 g/cm^2^) in both LEO (ISS ~ 400 km) and deep space (~ 1 astronomical unit (AU)) using NASA’s web-based OLTARIS platform; (ii) Utilize an updated analytic microdosimetric model to calculate the $y$-spectrum in a 1 μm liquid water sphere, for the individual GCR particles in the energy range of 1 MeV/amu—1 GeV/amu; (iii) Based on the above steps, calculate the average $Q$-values of GCR using a modified version of the microdosimetric TDRA methodology for both the LEO and deep space mission scenarios; (iv) Compare the present results for the mission $Q$-values against the ICRP- and NASA-based predictions, as well as against real measurements of $Q$ carried out in ISS and Space Shuttles (LEO) and by the MSL-RAD in deep space.

## Materials and methods

### Lineal energy ($\mathbf{y}$)

Microdosimetric quantities are important when studying the interaction of radiation with matter at sufficient small volumes of μm down to nm, in order to account for energy-loss straggling and the finite range of the liberated secondary electrons (*δ*-rays) (Rossi and Zaider 2012). In the context of microdosimetry, non-stochastic quantities, such us LET and absorbed dose, are replaced with their microdosimetric analogs of lineal energy $$(y)$$ and specific energy$$(z)$$, respectively. Lineal energy is the energy imparted (*ε*$$)$$ to the matter by a single energy-deposition event, which consists of statistically correlated depositions of energy as, for example, those by high energy particles and/or their secondary electrons divided by the mean chord length $$(\overline{l })$$ of the volume under study (International Commission on Radiation Units and Measurements 1983, 2011; Rossi and Zaider 2012):

1 $$y=\frac{\varepsilon }{\overline{l} }$$

For a sphere $\overline{l }=\frac{2}{3}d,$ where $d$ is the sphere’s diameter. It is evident from Eq. (1) that $y$ depends not only on the energy deposition, which is subject to radiation quality, but also on the size (and shape) of the volume. Because of the inherent statistical nature of the energy deposition process, each radiation quality is described by a probability density function $f(y)$. As a result, one can define two mean values of $y$, namely the frequency-mean lineal energy ${(y}_{\mathrm{F}})$ and the dose-mean lineal energy ${(y}_{\mathrm{D}})$ (Rossi and Zaider 2012):

2 $${y}_{\mathrm{F}}=\int yf\left(y\right)dy$$

3 $${y}_{\mathrm{D}}=\frac{1}{{y}_{\mathrm{F}}}\int {y}^{2}f\left(y\right)dy$$

For many radiobiological effects (including cancer-related effects), the RBE (or $Q$) increases with LET up to a certain value, beyond which any additional increase of LET causes a reduction of the biological effect (the so-called overkill effect Joint Task Group on Radiation Protection Quantities et al. 1986; Rossi and Zaider 2012)). This is particularly relevant to the heavy-ion component of the GCR owing to their very high LET values. Therefore, a saturated value, ${y}^{*}$, has been defined in ICRU Report 40 (Joint Task Group on Radiation Protection Quantities et al. 1986) which, for sphere diameters less than or equal to 1 μm, is expressed as:

4 $${y}^{*}=({125}^{2}/y)[1-{e}^{{-\left(y/125\right)}^{2}}]$$

### Generalized microdosimetric model

Ions interacting with matter in microscopic volumes will deposit their energy in a discrete manner that depends on the path traveled by the ion, their single-collision energy-loss spectrum, as well as the transport of δ-rays. Ions passing through the microscopic volume are characterized as ‘direct’ events (Xapsos et al. 1994). However, they can also deposit energy in the volume even if they miss and pass outside of it, through their δ-rays. These are termed ‘indirect’ events (Xapsos et al. 1994). A general-purpose analytical microdosimetric model to account for both direct and indirect events has been developed by Xapsos in a series of papers (Xapsos et al. 1994, 1996; Badavi et al. 2008; Papadopoulos et al. 2022), and successfully applied for calculating the energy deposition to Tissue Equivalent Proportional Counters (TEPCs) by space radiation (Shinn et al. 1999; Badavi et al. 2008, 2009). TEPCs (or Rossi counters) can directly measure $y$-spectra in a fixed volume that simulates microscopic human tissue. TEPCs have been exploited for radiation quality estimates in the ISS and various Space Shuttle missions (Badhwar et al. 1992b, a; Doke et al. 2001a).

A combination of these models with parameters deduced from Monte Carlo simulations with the Geant4-DNA toolkit (Incerti et al. 2010; Kyriakou et al. 2021) using updated physical models for the ionization and excitation cross sections of low-energy electrons in liquid water (Kyriakou et al. 2015, 2016) has been recently published (Papadopoulos et al. 2022). The electron parameters and the material constants needed for the model have been all deduced for liquid water, whereas the original model (Xapsos et al. 1994, 1996) employs water vapor-based values.

The combined microdosimetric model is briefly discussed below. The mean energy lost by ions and retained in the target volume is proportional to the ion’s LET and the mean chord length ($\overline{l }$) (Xapsos et al. 1994):

5 $$ \overline{E} = f_{{{\text{ion}}}} \times LET \times \overline{l}, $$

where $\overline{l }$ is the mean path length of the ion inside the volume and ${f}_{\mathrm{ion}}$ is the fraction of the energy loss of the primary particle that is retained within the volume calculated from (Xapsos 1992):

6 $$ f_{{{\text{ion}}}} = \frac{{{\text{ln}}\left[ {\frac{{T_{{{\text{el}},{\text{max}}}} \left( {\Delta + \Delta 1 + \Delta 2} \right)}}{{I^{2} }}{ }} \right]}}{{2{\text{ln}}\left[ {\frac{{T_{{{\text{el}},{\text{max}}}} }}{I}} \right]}}, $$

where $I$ is the mean excitation energy of the medium (International Commission on Radiation Units and Measurements 2014), ${T}_{\mathrm{el},\mathrm{max}}$ (keV) is the maximum energy of *δ*-rays, ${T}_{\mathrm{el},\mathrm{max}}=2.179 T$ (Xapsos et al. 1994), where $T$ is the ion’s kinetic energy in MeV/amu and $\Delta$ (keV) is the δ-ray energy whose range equals the mean chord length ($\overline{l }$) of the site:

7 $${R}_{\mathrm{el}}\left({T}_{\mathrm{el}}=\Delta \right)=\overline{l }$$

where ${R}_{\mathrm{el}}$ and ${T}_{\mathrm{el}}$ are the δ-ray range and kinetic energy, respectively. $\Delta 1$ corresponds to the energy loss of *δ*-rays (which are generated inside the volume but nevertheless escape it) that is retained in the volume. $\Delta 2$ accounts for energy transfers to atoms experiencing excitation or ionization by primary ions and subsequently produce δ-rays that escape the volume.

It can be shown that (Xapsos 1992; Xapsos et al. 1994, 1996):

8 $$\Delta 1+\Delta 2=\left(1-\frac{\Delta }{{T}_{el,max}}\right)(I+\Delta )$$

Energy-loss straggling is important for calculating the ionization spectrum of the ion in (sub)-cellular-sized volumes. Specifically, the probability density function, ${p}_{x,l}$ for a single particle traversal with energy deposition ($x$) and path length $l$, can be expressed as a log-normal distribution (Xapsos et al. 1996):

9 $$ p_{x,l} = \frac{1}{{\sqrt {2\pi } \sigma_{l} x}}Exp\left[ { - \left( {\frac{{\left( {ln\left( x \right) - \mu_{l} } \right)}}{{\sqrt 2 \sigma_{l} }}} \right)^{2} } \right], $$

where ${\sigma }_{l}$ and ${\mu }_{l}$ are related to the variance and the mean of the energy deposition, respectively, described by the following equations (Xapsos et al. 1996):

10 $$ \sigma_{l} = \sqrt {{\text{ln}}\left( {1 + V} \right)} , $$

11 $${\mu }_{l}=\mathit{ln}\left(\overline{E }\right)-0.5{\sigma }_{l}^{2}$$

$\overline{E }$ is the mean energy deposited to the site by the ion calculated from Eq. (5) for the mean chord length $\overline{l }$ and $V$ is the relative variance of random processes, such as path length, energy-loss straggling, and LET fluctuations (Rossi and Zaider 2012). Single energy ions, however, are considered to have no significant LET fluctuations. For energy depositions of a single ion event, relative variance can be calculated from (Xapsos et al. 1996):

12 $$ V = V_{{{\text{str}}}} + V_{l} , $$

where ${V}_{l}$ is the relative variance of the path length, which is equal to $1/8$ (Kellerer 1985; Xapsos et al. 1996; Rossi and Zaider 2012). ${V}_{\mathrm{str}}=\overline{{\delta  }_{2}}/\overline{E }$ is the relative variance of energy-loss straggling.$\overline{{\delta  }_{2}}$ is the dose-mean of the energy deposited after the ion–electron interaction. For small energy depositions, it can be approximated by the following equation (Xapsos et al. 1994):

13 $$ \overline{{\delta_{2} }} = A{ }\Delta^{\rm B} , $$

where *A*, *B* are material-related constants. The methodology for calculating the A and B parameters can be found in (Xapsos et al. 1996). *A*, *B* values differ for direct (ion), indirect (electron) events, and the respective material and can be obtained for liquid water (Papadopoulos et al. 2022) or for water vapor (Xapsos et al. 1994, 1996).

Eventually, the probability density function of producing energy depositions ($x$) is given from ${f}_{x}$, which is the convolution of the energy-straggling process ${p}_{x,l}$ and the chord length distribution ${c}_{l}=2l/{d}^{2}$ of the primary ion (Xapsos et al. 1996):

14 $${f}_{x}=\int {p}_{x,l}{c}_{l}ds$$

For electron events, the same procedure with direct events can be followed, using Eqs. (9)–(14) with some modifications in Eqs. (5), (11), and (12). Secondary electrons with mean LET value, ${LET}_{e}$, traversing the volume with path length $l$, deposit their energy in the volume in a manner similar to ions (Xapsos et al. 1994; Badavi et al. 2009):

15 $${\overline{E} }_{\mathrm{el}}={LET}_{e}\times l$$

It is assumed that the secondary electron energy loss is retained into the site, because higher electron generations (tertiary, quaternary, etc.) are rarely energetic enough to escape the volume, so a parameter $f$ (Eq. (6)) is not needed for indirect events. Relative variance of indirect events, calculated from Eq. (12) is now changed in order to account for the electron LET fluctuations (Xapsos et al. 1996; Badavi et al. 2009):

16 $$V={V}_{\mathrm{str}}+{V}_{l}+{V}_{\mathrm{LET}}$$

$\overline{{\delta  }_{2}}$ for indirect events is calculated from Eq. (13) with different *A*, *B* values from those of the direct events (Xapsos et al. 1994; Papadopoulos et al. 2022).

The last thing to combine before calculating the total energy deposition, is the probability of an energy deposition to occur from a direct ($P$) or indirect $(1-P)$ event. The fraction of indirect events is given by the following expression (Olko 2006; Badavi et al. 2009):

17 $$ \left( {1 - P} \right) = \frac{{\left( {1 - f_{{{\text{ion}}}} } \right)\overline{E}_{{{\text{ion}}}} }}{{f_{{{\text{ion}}}} \overline{E}_{{{\text{el}}}} + \left( {1 - f_{{{\text{ion}}}} } \right)\overline{E}_{{{\text{ion}}}} }}, $$

${f}_{\mathrm{ion}}$ is calculated from Eq. (6), while the mean energy that is deposited in the site from ions (${\overline{E} }_{\mathrm{ion}}$) and electrons (${\overline{E} }_{\mathrm{el}}$) is calculated from Eqs. (5) and (15) accordingly by setting $l=\overline{l }=2d/3$.

The combined ion and electron energy distributions can then be calculated from (Xapsos et al. 1994; Olko 2006; Badavi et al. 2009):

18 $$ f_{{x,{\text{total}}}} = Pf_{{x,{\text{ion}}}} + \left( {1 - P} \right)f_{{x,{\text{ el}}}} , $$

where ${f}_{x,ion}$ and ${f}_{x, el}$ are the ion and electron energy probability density functions obtained from Eq. (14).

### Methods for calculating the Quality Factor ($\mathbf{Q}$)

#### ICRP Report 60

The International Commission on Radiological Protection (ICRP) Publication 60 (International Commission on Radiological Protection 1991) has defined $Q$ as a continuous function of the unrestricted Linear Energy Transfer (LET, $L$) in water. The simplicity of the $Q(L)$ approach along with the availability of an analytic formula for calculating *L* (i.e., Bethe’s stopping-power formula) for ions over a broad energy range is the main advantage of this method. According to ICRP, the $Q\left(L\right)$ values can be obtained from the following equations (International Commission on Radiological Protection 1991):

19 $$Q\left(L\right)=1, L<10\mathrm{keV}/\mathrm{\mu m }$$

20 $$ Q\left( L \right) = 0.32L - 2.2{ },10{\text{ keV}}/{\mu m} \le L \le { }100{\text{ keV}}/{\mu m} $$

21 $$Q\left(L\right)=\frac{300}{\sqrt{L}}, L>100\mathrm{ keV}/\mathrm{\mu m}$$

The above $Q(L)$ equations (Eqs. 19–21) have been deduced from RBE data based on animal experiments and radiobiological studies at cellular level (International Commission on Radiological Protection 1991).

#### Theory of dual radiation action model

The most practical (and used) formulation of the Theory of Dual Radiation Action (TRDA) is the so-called “site model” which assumes that cellular biological effects are caused by the pair-wise interaction of sub-lesions produced within a fixed-size target. Importantly, the probability of two sub-lesions to interact is independent of their geometric distribution within the site. It follows from TDRA that sub-lesions are produced either in the same track (for high-LET radiation) or in two separate tracks (for low-LET radiation). The general expression of RBE of the site version of TDRA takes the following form (Kellerer and Rossi 1978; Rossi and Zaider 2012):

22 $${\mathrm{RBE}}_{\mathrm{TDRA}}=\frac{\sqrt{{{(c\times y}_{D,L})}^{2}+4{D}_{H}({c\times y}_{D,H}+{D}_{H})}-{(c \times y}_{D,L})}{2{D}_{H}}$$

where *c* is a normalization constant (Kyriakou et al. 2021), ${D}_{H}$ is the dose from the high-LET radiation and ${y}_{D,L}$, ${y}_{D,H}$ are the low- and high-LET dose-mean lineal energy, respectively. Then, in the low-dose regime (${D}_{H}\ll {c \times y}_{D,L}$) where $Q={RBE}_{D\to 0}$, Eq. (22) reduces to:

23 $${Q}_{\mathrm{TDRA}}=\frac{{y}_{D,\mathrm{test}}}{{y}_{D,\mathrm{ref}}}$$

where ${y}_{D,\mathrm{test}}$ and ${y}_{D,\mathrm{ref}}$ are the dose-mean lineal energy of the test and the reference radiation, respectively. For the high-LET particles of GCR, ${y}_{D}$ is replaced by the dose-weighted saturated lineal energy (${y}_{D}^{*}$) (Joint Task Group on Radiation Protection Quantities et al. 1986). Hence, Eq. (23) becomes (Joint Task Group on Radiation Protection Quantities et al. 1986; Rossi and Zaider 2012):

24 $${Q}_{\mathrm{TDRA}}=\frac{{y}_{D,\mathrm{test}}^{*}}{{y}_{D,\mathrm{ref}}^{*}}$$

#### NASA model

NASA has developed a radiation cancer-risk model that distinguishes the RBE for solid cancers and leukemia based on recent radiobiological and epidemiological data. It also accounts for the different ionization density contributions of particles using the track structure parameter ${Z}^{2}/{\beta }^{2}$ and separates between the low-LET component (${Q}_{\mathrm{Low}}$) and the high-LET (${Q}_{\mathrm{High}})$ component. Contrary to the LET approximation of ICRP, NASA characterizes $Q$ with a fluence-based approximation of risk cross sections, $\Sigma ({\rm Z},{\rm E})$. Risk cross section is simply the probability per unit fluence of a biological effect (e.g., leukemia) to occur for a specific ion with atomic number $Z$ and energy $E$ and it is based on the biophysical model of Katz (Katz et al. 1971). The NASA model for $Q$ is described by Eqs. (25) and (26) (Cucinotta et al. 2011):

25 $$ Q_{{{\text{NASA}}}} = \left( {1 - P\left( {Z,E} \right)} \right) + \frac{{6.24(\Sigma_{o} /\alpha_{\gamma } )}}{{{\text{LET}}}}P\left( {Z,E} \right), $$

where

26 $$P\left(Z,E\right){=\left(1-{e}^{\frac{{-({\rm Z}^*/\beta )}^{2}}{k}}\right)}^{m}(1-{e}^{-(E/0.2)})$$

The first term of the right-hand of Eq. (25), is the ${Q}_{\mathrm{Low}}$, while the second term accounts for ${Q}_{\mathrm{High}}$. The ratio $({\Sigma }_{0}/{\alpha }_{\gamma })$ is treated as a fitting parameter and ${\alpha }_{\gamma }$ is the linear slope of the dose–response curve of γ-rays (low-LET). ${Z}^{*}$ is the effective charge, *β* is the velocity of the particle normalized to the speed of light, and the term $(E/0.2)$ accounts for the reduced effectiveness (reduced radial dimensions) of the particles as they slow down. The experimental parameters $k$ and $m$ account for the location of the maximum of the RBE including the saturation effects, and the slope of the cross section, ${\Sigma }_{0}$, respectively. The central/standard values for the above parameters obtained from NASA’s 2012 cancer risk model (Cucinotta et al. 2011), are shown in Table 1. Additional modifications of NASA’s model and updates can be found in literature (Borak et al. 2014; Cucinotta et al. 2020a).

**Table 1:** Standard values of the fitting parameters used in the NASA model for *Q*

Fitting parameters	Solid cancer	Leukemia
$({\Sigma }_{0}/{\alpha }_{\gamma })$	7,000/6.24	1,750/6.24
$k$	1000, Z ≤ 4	1000, Z ≤ 4
	500, Z > 4	500, Z > 4
$m$	3	3

#### Mission quality factor

The mission $Q$-value (GCR contribution) was determined from the integration of each ion ${Q}_{Z}(E)$ over the entire energy spectrum and weighted by their contribution to the total dose. It was then summed for all ion charges from *Z* = 1 to *Z* = 26. It is calculated according to Eq. 27:

27 $$ \overline{Q}_{{{\text{GCR}}}} = \frac{{\mathop \sum \nolimits_{Z} \mathop \smallint \nolimits_{{E{\text{min}}}}^{{E{\text{max}}}} Q_{Z} \left( E \right){ }D_{Z} \left( E \right)dE}}{{\mathop \sum \nolimits_{Z} \mathop \smallint \nolimits_{{E{\text{min}}}}^{{E{\text{max}}}} D_{Z} \left( E \right)dE}} $$

where ${Q}_{Z}(E)$ is the quality factor of an ion with charge *Z* and energy *E* calculated by one of the three methodologies examined in this work (namely, ICRP-60 Eqs. (19–21)), TDRA Eqs. (23–24), and NASA model Eqs. (25–26)), ${D}_{Z}(E)$ is the corresponding absorbed dose ($\approx f\mathrm{lux}\times \mathrm{LET})$ of each ion with energy $E$, and the limits of integration are set at $E\mathrm{min}=1 \mathrm{MeV}$ and $E\mathrm{max}=1 \mathrm{GeV}$.

### OLTARIS software

For the assessment of the radiation environment in space and the calculation of the average $Q$ for specific missions, NASA has developed the online platform OLTARIS (Singleterry et al. 2011). Two space environments have been examined in the present work. The first was studied inside Earth’s magnetic field, in a circular LEO with altitude 400 km and inclination 51.6°, simulating the orbit of the International Space Station (ISS). The second was an orbit at deep space (1 AU). The GCR spectrum in an extended energy range of 1 MeV/amu- 1 GeV/amu has been calculated in this work using the Badhwar-O’Neill 2020 model (Slaba and Whitman 2020) (incorporated to OLTARIS), using 1977 Solar Minimum conditions. The initial spectrum of the GCR both for LEO (ISS) and deep space that was transported through aluminum shielding was 1 MeV/amu—1,000 GeV/amu, which is the default energy range of the OLTARIS platform. After transportation and for the estimation of the $Q$-values, the energy range of 1 MeV/amu–1 GeV/amu was used since the analytical microdosimetric model used for calculating the ${y}_{D}$ values does not extend to energies greater than 1 GeV/amu. This limitation affected the calculations by less than 2–3%. This was deduced by extending the calculations to ion energies beyond 1 GeV/amu assuming a constant ${y}_{D}$ value equal to that at 1 GeV/amu (since this assumption overestimates the true ${y}_{D}$ beyond 1 GeV/amu, it offers an upper limit to the error made in our $Q$-value by cutting the spectrum at 1 GeV/amu). The $Q$-values have been calculated for each ion without its isotopes in order to be consistent with the LET calculations. GCR flux from 1 MeV/amu—1 GeV/amu has been obtained behind aluminum thicknesses of 0 g/cm^2^ to 30 g/cm^2^ — both for LEO (ISS orbit) and deep space (1 AU). Table 2 shows the orbital characteristics of the specific mission scenarios considered in this work.

**Table 2:** Orbital parameters for the two different space scenarios used in OLTARIS

Orbital parameters	Low earth orbit (ISS)	Deep space
Altitude	~ 400 km	~ 1 AU
Inclination	~ 51.6°	–
Solar cycle	1977 Solar minimum	1977 Solar minimum
Model	GCR B-ON 2020	GCR B-ON 2020
Shielding	Aluminum 0–30 g/cm^2^	Aluminum 0–30 g/cm^2^

## Results

**Fig. 1:**
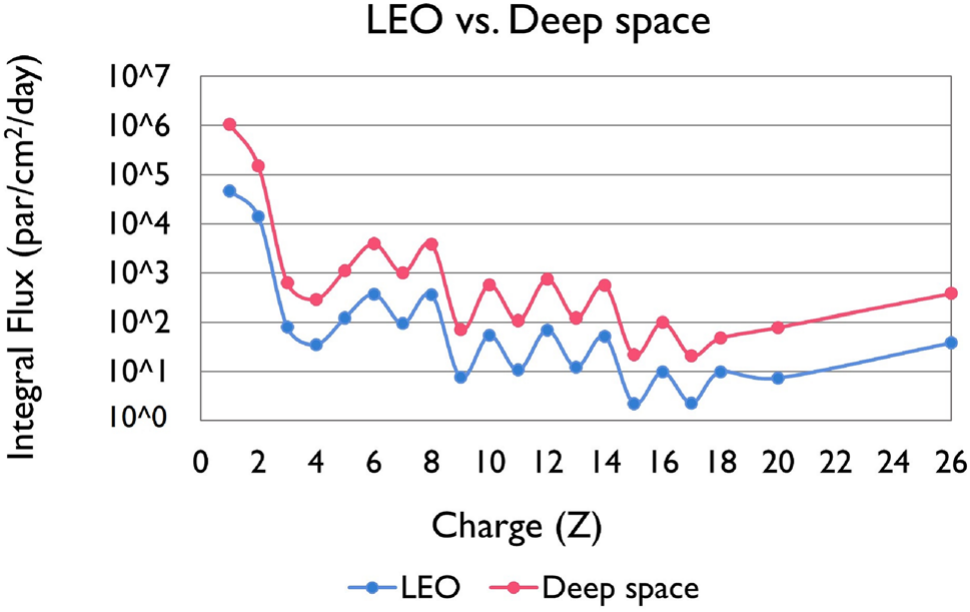
Integral Flux (particles/cm^2^/day) of Galactic Cosmic Ray (GCR) particles from protons up to $Fe$, obtained from the OLTARIS platform for 1977 Solar Minimum conditions. Red line represents the integral flux in deep space (1 AU) and blue line the integral flux in Low Earth Orbit (LEO) (International Space Station (ISS) ~ 400 km), both for no shielding conditions

**Fig. 2:**
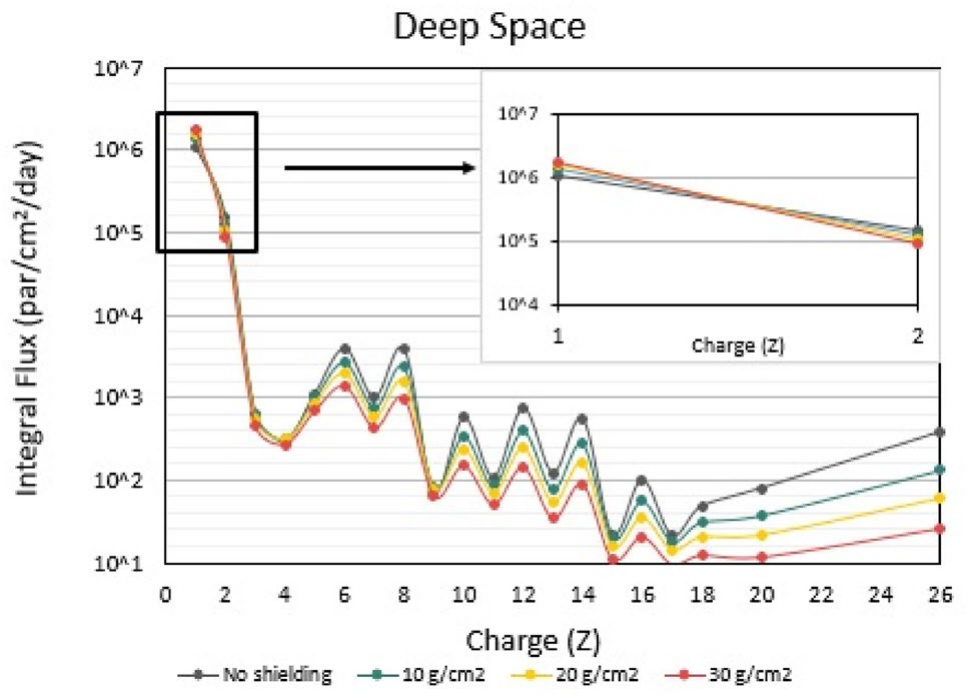
Integral Flux (particles/cm^2^/day) of Galactic Cosmic Ray (GCR) particles from protons up to $Fe$ for deep space, obtained from the OLTARIS platform for 1977 Solar Minimum conditions. The calculations were made for no shielding conditions, and for 10, 20, and 30 g/cm^2^ aluminum shielding. The embedded figure represents the integral flux for Z = 1–2 for different aluminum shielding

**Fig. 3:**
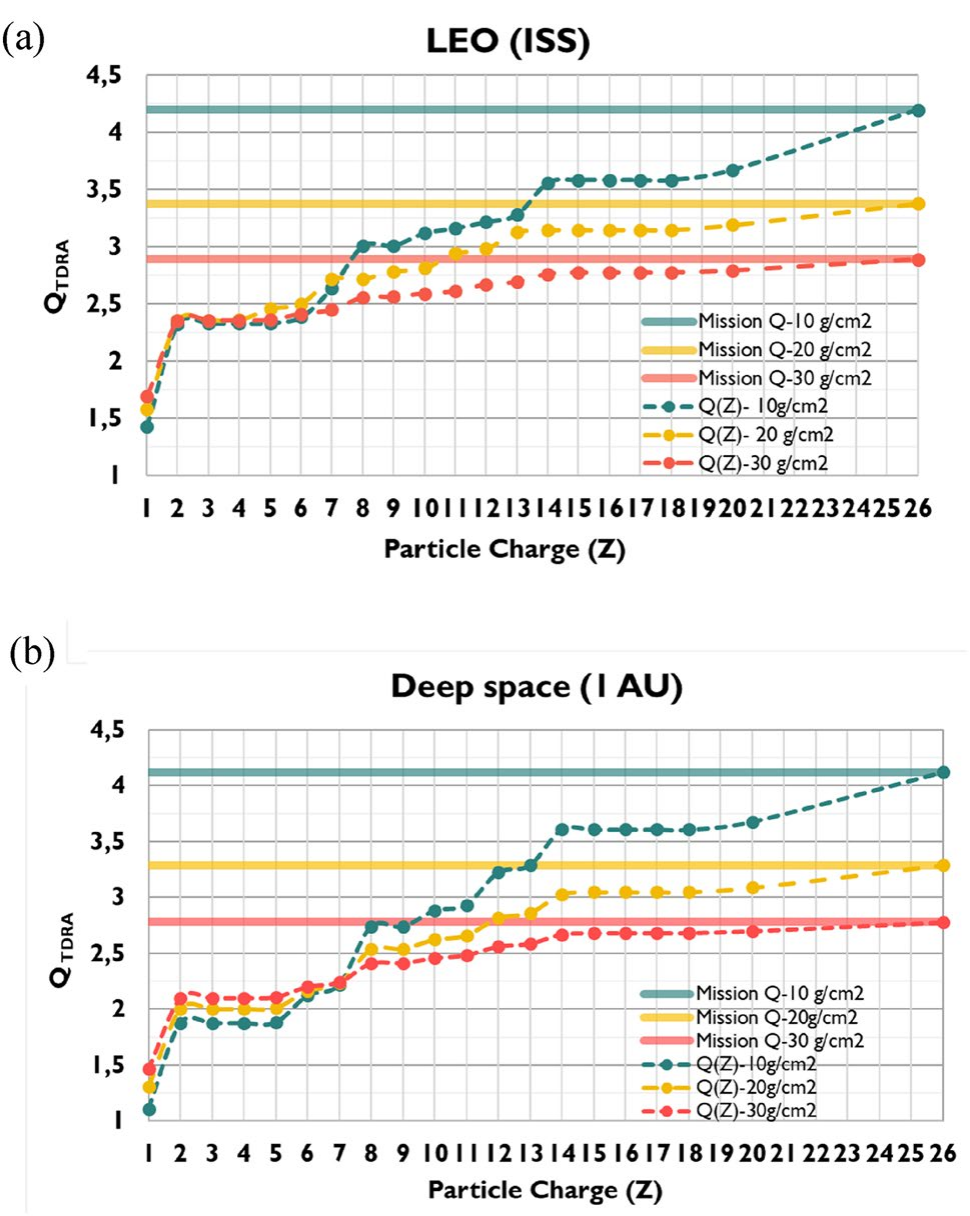
Cumulative $Q$-value of Galactic Cosmic Ray (GCR) as a function of particle’s charge ($Z$) (dot-lines), calculated by the Theory of Dual Radiation Action (TDRA) for (**a**) Low Earth Orbit (LEO) (International Space Station (ISS) ~ 400 km) and (**b**) deep space (1 AU), both calculated behind aluminum shielding of 10–30 g/cm^2^. Thick, solid lines are the mission *Q*-values

The average quality factors of GCR for the mission were calculated using a modified version of the TDRA methodology (Eq. (24)), including all ions from protons up to Argon ($Ar$) and Iron ($Fe$). The saturated dose-mean lineal energy (${y}_{D}^{*}$) values were calculated from a combined version of the microdosimetric models of Xapsos et al. 1994, 1996 with updated physical parameters (Papadopoulos et al. 2022). The ${y}_{D}^{*}$ and subsequently $Q$-values have been determined for 1 μm sphere diameter of liquid water which is the sphere size of relevance to experimental measurements with TEPCs (e.g., in ISS). As reference radiation ($Q$≡1), protons at 100 MeV were used since they can be safely considered as a low-LET radiation ($\mathrm{LET}\approx 0.73$ keV/μm). The GCR spectrum is obtained from the web-based OLTARIS platform for a deep space (1 AU) and LEO orbit (ISS) in the energy range of 1 MeV/amu- 1 GeV/amu. Figure 1 depicts the integral fluxes (particles/cm^2^/day) of particles from protons up to $Ar$ and $Fe$, with no-shielding conditions. The integral flux (particle/cm^2^/day) for deep space and Z = 1–26 particles is depicted in Fig. 2. The integral fluxes were calculated for no shielding conditions, as well as for 10, 20 and 30 g/cm^2^ aluminum shielding using the OLTARIS platform (HZETRN transport code). The inset figure shows the integral fluxes of Z = 1–2, in order to observe the rise of Z = 1 particles with increasing shielding. Figure 3 shows the cumulative $Q(Z)$-value (${Q}_{H},{Q}_{H}+{Q}_{He}$,…) (dot-lines) for (a) LEO (ISS) and (b) deep space missions as a function of particle’s charge ($1\le Z\le 26)$, as well as the mission (total) *Q*-value (thick, solid lines), both calculated by the modified TDRA approach, behind different aluminum shielding (10–30 g/cm^2^) and 1977 solar minimum conditions. Aluminum shielding of this range covers most of the nominal shielding values used in spacecrafts for space missions (Space Transportation System, ISS).

**Fig. 4:**
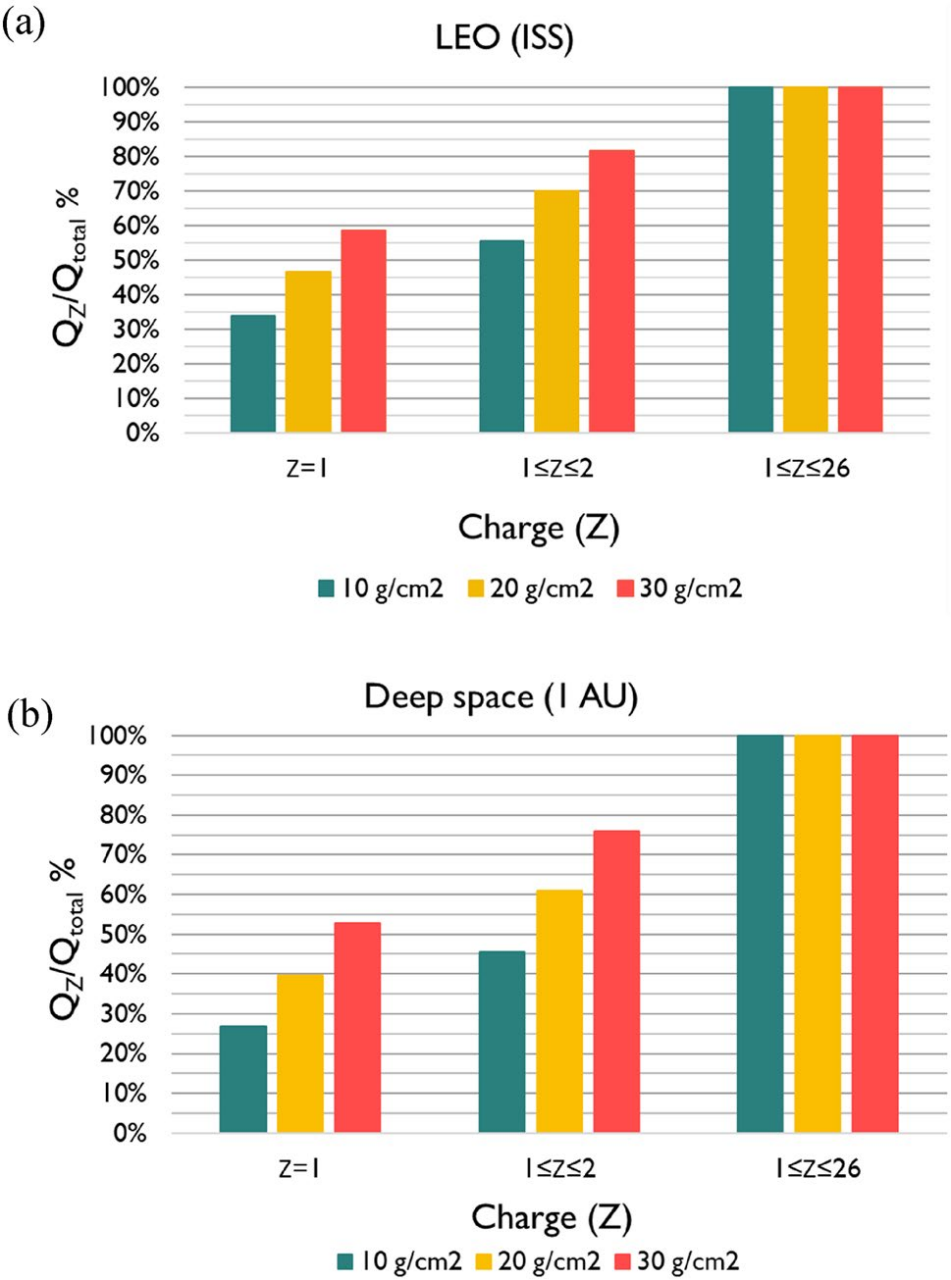
Contribution (%) of different Galactic Cosmic Ray (GCR) particles to the total (mission) $Q$-value calculated with the Theory of Dual Radiation Action (TDRA) methodology for (**a**) Low Earth Orbit (LEO) (International Space Station (ISS) ~ 400 km) and (**b**) deep space (1 AU), both behind aluminum shielding of 10–30 g/cm.^2^

In addition to mission $Q$-values, it is also useful to investigate the contribution of each particle relative to the total $Q$-value, in order to further understand the impact of the space radiation environment to the carcinogenic risk to astronauts. Figure 4, depicts the cumulative contribution (in %) of the different GCR particles, relative to the total (mission) $Q$ (i.e., ${Q}_{H}/{Q}_{\mathrm{total}},({Q}_{H}+{Q}_{He})/{Q}_{\mathrm{total}},\dots )$, for (a) LEO (ISS) and (b) deep space mission scenarios, both calculated behind different aluminum shieldings $(10-30$ g/cm^2^). For better insight, the results are grouped into GCR particles of different atomic number (*Z* = 1, *Z* = 1–2, *Z* = 1–26).

**Fig. 5:**
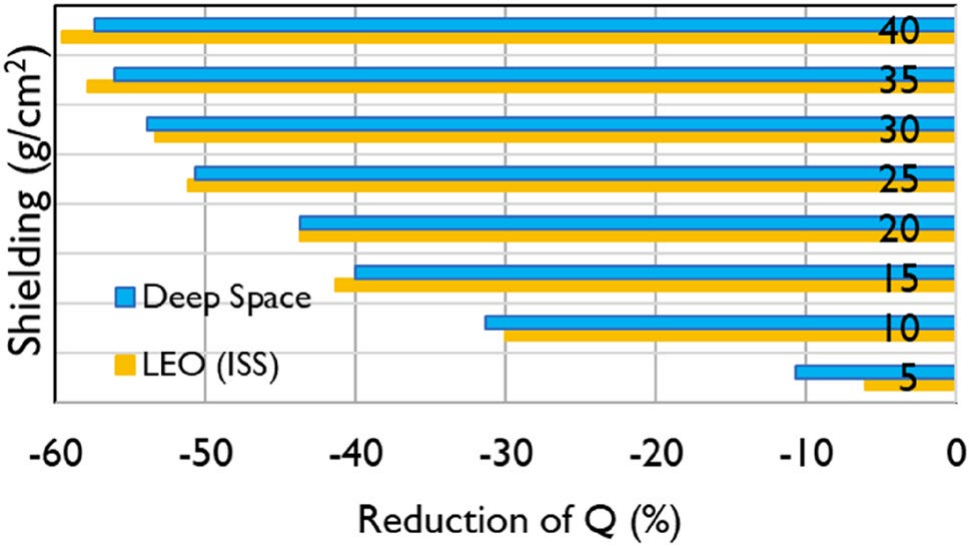
Reduction (%) of the Theory of Dual Radiation Action (TDRA)-based mission $Q$-value, with increasing aluminum shielding (5–40 g/cm^2^) for Low Earth Orbit (LEO) (International Space Station (ISS) ~ 400 km) and deep space (1 AU)

The influence of shielding on the mission quality factor is depicted in Fig. 5 for both LEO and deep space.

In Tables 3 and 4, the present mission $Q$-values calculated by the TDRA approach (using a 1 μm liquid water target sphere), are compared against NASA’s model (${Q}_{\mathrm{NASA}})$ and the ICRP Report 60, $Q(L)$. Comparisons were made for LEO (ISS ~ 400 km) and deep space (1 AU), for different aluminum shielding thickness in the range of $10-30$ g/cm^2^.

**Table 3:** Low Earth Orbit (LEO) (International Space Station (ISS), ~ 400 km) mission $Q$-values calculated by the present Theory of Dual Radiation Action (TDRA) approach are compared against $Q$-values calculated according to ICRP Report 60 (ICRP 1991) and the NASA model (Cucinotta et al. 2011)

LEO (ISS)	This work (*Q*- values from TDRA)	ICRP report 60 ($Q$-values from OLTARIS)	Difference (%) ICRP as baseline
Al (g/cm^2^)			
10	4.2	4.6	−8.7
20	3.38	3.14	5.79
30	2.89	2.64	9.47

LEO (ISS)	This work ($Q$-values from TDRA)	NASA model ($Q$-values from OLTARIS)	Difference (%) NASA as baseline
Al (g/cm^2^)			
10	4.2	4.2	0
20	3.38	3.27	3.37
30	2.89	2.91	−0.68

**Table 4:** Deep space (1 AU) mission $Q$-values calculated by the present version of the Theory of Dual Radiation Action (TDRA) approach are compared against $Q$-values calculated according to ICRP Report 60 (ICRP 1991) and the NASA model (Cucinotta et al. 2011)

Deep space (1 AU)	This work ($Q$-values from TDRA)	ICRP report 60 ($Q$-values from OLTARIS)	Difference (%) ICRP as baseline
Al (g/cm^2^)			
0	5.95	6.22	−4.3
10	4.12	3.9	5.64
20	3.29	2.89	13.84
30	2.78	2.51	10.76

Deep space (1 AU)	This work ($Q$-values from TDRA)	NASA model ($Q$-values from OLTARIS)	Difference (%) NASA as baseline
Al (g/cm^2^)			
0	5.95	6.32	−5.85
10	4.12	4.19	−1.67
20	3.29	3.2	2.81
30	2.78	2.86	−2.8

Additionally, in Tables 5 and 6, the present mission $Q$-values calculated by the TDRA approach (using a 1 μm liquid water target sphere) are compared against measurements from active detectors (TEPCs) that have flown in ISS and Space Shuttle (Table 5) as well as aboard the MSL-RAD during the period of its transit to Mars with Curiosity (Table 6).

**Table 5:** Low Earth Orbit (LEO) (International Space Station (ISS) ~ 400 km) mission $Q$-values calculated by the present Theory of Dual Radiation Action (TDRA) approach are compared against measured $Q$-values by Tissue Equivalent Proportional Counters (TEPCs) aboard ISS (Reitz et al. 2005) and Space Shuttle missions (Beaujean et al. 1999; Doke et al. 2001a; Badhwar 2002; Badhwar et al. 2002). Shielding values presented are the nominal values

LEO (ISS)	This work ($Q$-values from TDRA)	Space shuttle (ISS orbits)	ISS
Al (g/cm^2^)			
~ 10	4.2	2.97 ≤ $Q$ ≤ 4.33	$-$
~ 20	3.38	$-$	2.8 ≤ $Q$ ≤ 3.7

**Table 6:** Deep space (1 AU) mission $Q$-values calculated by the present Theory of Dual Radiation Action (TDRA) approach are compared against measured $Q$-values by Tissue Equivalent Proportional Counters (TEPCs) aboard the MSL-RAD (Hassler et al. 2014)

Deep space (1 AU)	This work ($Q$-values from TDRA)	MSL RAD (Cruise to Mars)
Al (g/cm^2^)		
10–30	2.78 ≤ $Q$ ≤ 4.12	3.84 ± 0.25

## Discussion

It is clear from Fig. 1 that even-numbered high-Z particles (such as $$C$$,$$O,Mg,Si,Fe)$$ are more abundant than odd-numbered particles. The elemental composition of GCR provides useful insight into their origin. The propagation of elements into the interstellar gas, the nuclear interactions, the acceleration mechanisms, and the first ionization potential, are the key parameters governing their abundance. The reason lighter elements from *Li* to *B* (Z = 3–5) are relatively more abundant than heavier particles is due to the interaction of the heavier source particles, such as carbon, oxygen, or nitrogen with the interstellar gas as they propagate into the heliosphere and break into these lighter charged particles. For 5 < Z < 26, the pairing effect (greater binding energies) is the underlying reason for the greater abundances of the even-numbered than odd-numbered charged particles (Simpson 1983; Straume et al 2017). Furthermore, the absorbed doses are much higher in deep space (1 AU) than in LEO (ISS ~ 400 km), since the GCR flux is significantly higher for all Z (Fig. 1). Figure 3 shows that mission $$Q$$-values for the GCR spectrum in both LEO and deep space missions are very similar and vary between 2.9 and 4.2 depending on shielding. The details of the cumulative distribution of $$Q$$ as a function of Z reflect the higher contribution of even-numbered Z particles relative to the odd-numbered particles, while it is slightly more pronounced for deep space than for LEO. As expected, the effect of shielding is significant for both LEO and deep space, specifically within the range of aluminum shielding encountered in space missions (10–30 g/cm^2^)$$, $$$$Q$$ varies by a factor of ~1.5, i.e., from $$Q$$=2.9 (10 g/cm^2^) to $$Q$$=4.2 (30 g/cm^2^). An interesting observation is that with increasing shielding, the contribution of low-Z particles ($$1\le Z\le 2$$) is increased compared to the high-Z particles (Fig. 4), from 55 to 80% for LEO and from 45 to 75% for deep space. This stems from the fact that, with increasing shielding, more high-Z particles are stopped in the shielding, if their energy is relatively small, or undergo nuclear fragmentation. The latter interactions produce low-Z ions capable of penetrating the shielding material (Fig. 2).

Another interesting observation is that, as shown in Fig. 5, an increase of shielding from 10 to 30 g/cm^2^ (i.e., by a factor of 3) results in only a moderate reduction of $Q$ by 30% (10 g/cm^2^) to 50% (30 g/cm^2^), and this is true for both LEO and deep space. This may be explained by the increased production of low-Z fragments with increasing shielding (as also discussed above) which somewhat compensates for the higher absorption of the high-Z particles. For aluminum values greater than 30 g/cm^2^, the mission $Q$-values do not decrease significantly. For this reason, a shielding between 25 and 30 g/cm^2^ (especially for deep space missions) seems adequate if one considers the trade-off between increased shielding (weight, cost) and relative reduction of $Q$. The cost of aluminum shielding depends on several factors (e.g., market, method of production, and geometry configuration of spacecraft), and the decision on the shielding material and value would also include the reduction of absorbed doses and other engineering issues (Wilson et al. 1998; Sager 1992; Adams et al. 2005). It should also be noted that hydrogen-rich materials (polyethylene, lithium hydride, and water) may be more suitable than aluminum for radiation mitigation, as they have higher values of mass stopping power and are more efficient in stopping neutrons due to nuclear elastic scattering (Cucinotta et al. 2006; Durante 2014; Naito et al. 2020; Gohel and Makwana 2022). The present, TDRA-based $Q$-values are in good agreement with both the ICRP Report 60 Recommendations and NASA’s model (Tables 1 and 2) despite the quite different methodologies. Specifically, the TDRA-based $Q$-values are within ~ 14% (deep space) and ~ 10% (LEO) of the ICRP LET-based $Q$-values. Even better agreement was found between the TDRA-based $Q$-values and NASA’s model with differences up to 3% for both missions.

In hadron therapy, it is generally assumed that the discrepancies among the different methods for RBE calculations for deterministic effects should not exceed ~ 5%, due to the need of precise treatment planning in the tumor volume and organs at risk. However, in space radiation protection, the lack of suitable data for heavy ions regarding their low-dose RBE for stochastic effects, causes much larger uncertainties which, inevitably, affect the organ dose equivalent calculations for planned missions. As a result, differences between TDRA and ICRP-60 methodologies of 10–14% in mission $Q$-values may be considered modest. Nevertheless, $Q(Z)$ values averaged over the spectrum of the individual ions may deviate substantially among the different methodologies (TDRA, ICRP-60, NASA), which may be important when considering cancer risk evaluation of astronauts for specific ions. The differences in $Q$-values among the different methodologies of NASA, ICRP-60, and microdosimetry approaches, for protons and heavier ions have been further discussed in literature (Papadopoulos et al. 2022; Cucinotta et al. 2013; NCRP Report 153; International Commission on Radiological Protection 2013).

The present, TDRA-based $Q$-values are also within the range of measured $Q$-values (Tables 3 and 4). For LEO, measurement results were obtained from TEPCs and other active detectors that have flown onboard the Space Shuttle missions and ISS (~ 400 km). For 10 g$-20$/cm^2^ aluminum shielding, which is representative of the Space Shuttle and ISS average or median values for the locations of the detectors, the TDRA-based $Q$-values are between 3.4 and 4.2, which is well within the measured range (Table 3). However, thickness distributions have to be applied in order to estimate more realistic $Q$-values, since there are parts of spacecrafts that are less or heavier shielded. Various passive and active detectors are available for space missions toward radiation quality (e.g., LET spectra) measurements. Active detectors such as TEPCs, besides providing real-time read-out, are generally considered tissue-equivalent and, therefore, suitable for simulating energy deposition spectra in the human tissue (Parisi et al. 2022). However, TEPC measurements for simulated tissue volumes at the nanometer scale (diameter < 100 nm) are difficult and less reliable. For example, the Auger electron flux in tissue is smaller than in water, due to the smaller concentration of oxygen. This limits the ability of a TEPC gas (considered to be tissue equivalent) to simulate lineal energy spectra in liquid water material. As a result, Monte Carlo simulation techniques offer a valuable theoretical tool for obtaining microdosimetry spectra (e.g., y-spectra) at the cellular and DNA scale, toward understanding the biological effects of ions relevant to the space radiation environment (Kyriakou et al. 2021; Nikjoo et al. 2016; Lindborg and Nikjoo 2011). For deep space calculations with aluminum shielding in the range $10-30$ g/cm^2^, the TDRA-based $Q$-values vary between 2.8 and 4.1 which is close to the value of 3.8 measured by MSL-RAD in deep space (Table 4).

An advantage of the present microdosimetric approach is that it overcomes the physical shortcomings of LET-based approaches, which do not accurately account for the energy deposition process since they neglect its stochastic nature (i.e., energy-loss straggling) as well as the finite range of secondary electrons (δ-rays), both of which may be crucial for HZE particles (e.g., ICRP 1991). The central physical quantity of lineal energy is directly measurable by the active dosimeters (e.g., TEPCs) used in space missions (e.g., ISS, MSL-RAD). It is also worth emphasizing that the fully analytic form of the present approach facilitates predictive calculations of the average $Q$-values in different mission scenarios.

## Conclusion

A generalized analytical microdosimetric model that considers energy-loss straggling and δ-rays transport was utilized in order to calculate lineal energy spectra in 1 μm liquid water sphere that were subsequently used to determine the average GCR quality factor ($Q$) based on the TDRA methodology for two mission scenarios, namely an ISS orbit (LEO) and a deep space orbit (1 AU). The GCR spectra behind aluminum shielding in the range of $0-30$ g/cm^2^ for the above radiation environments were obtained from NASA’s online platform OLTARIS. These results were compared against the LET-based $Q$-values of ICRP and NASA’s $Q$ model. It was shown that the present results for the average $Q$-value of the GCR spectrum are in good agreement with both the ICRP and NASA model predictions for both mission scenarios. The present results are also within the range of values measured by TEPCs in both LEO (ISS, Space Shuttle) and deep space (MSL-RAD). An advantage of the present microdosimetric approach is that it overcomes the physical shortcomings of LET-based approaches (e.g., ICRP) while its central physical quantity (lineal energy) is directly measurable by the well-established active dosimeters (e.g., TEPC) that are widely used in space missions (e.g., ISS, MSL-RAD). Finally, it is worth emphasizing that the present approach is fully analytic and robust, thus, facilitating its practical use for predictive calculations of the average $Q$-values of different mission scenarios.

## Data Availability

The datasets generated during and/or analyzed during the current study are available from the corresponding author on reasonable request.
